# Transcriptome Sequencing of Peripheral Blood Mononuclear Cells from Elite Controller-Long Term Non Progressors

**DOI:** 10.1038/s41598-019-50642-x

**Published:** 2019-10-03

**Authors:** Francisco Díez-Fuertes, Humberto Erick De La Torre-Tarazona, Esther Calonge, Maria Pernas, María del Mar Alonso-Socas, Laura Capa, Javier García-Pérez, Anavaj Sakuntabhai, José Alcamí

**Affiliations:** 1 AIDS Immunopathology Unit, Centro Nacional de Microbiología, Instituto de Salud Carlos III, Ctra. Majadahonda-Pozuelo, Km. 2, 28220 Majadahonda, Madrid Spain; 2 Hospital Clínic-Institut d’Investigacions Biomèdiques August Pi i Sunyer (IDIBAPS), 08036 Barcelona, Spain; 3 Molecular Virology Unit, Centro Nacional de Microbiología, Instituto de Salud Carlos III, Ctra. Majadahonda-Pozuelo, Km. 2, 28220 Majadahonda, Madrid Spain; 4 Servicio de infecciones, Hospital Universitario de Canarias, 38320 Santa Cruz de Tenerife, Spain; 5 Functional Genetics of Infectious Diseases, Pasteur Institute, 75015 Paris, France

**Keywords:** Retrovirus, Transcriptomics

## Abstract

The elite controller (EC)-long term non-progressor (LTNP) phenotype represent a spontaneous and advantageous model of HIV-1 control in the absence of therapy. The transcriptome of peripheral blood mononuclear cells (PBMCs) collected from EC-LTNPs was sequenced by RNA-Seq and compared with the transcriptomes from other phenotypes of disease progression. The transcript abundance estimation combined with the use of supervised classification algorithms allowed the selection of 20 genes and pseudogenes, mainly involved in interferon-regulated antiviral mechanisms and cell machineries of transcription and translation, as the best predictive genes of disease progression. Differential expression analyses between phenotypes showed an altered calcium homeostasis in EC-LTNPs evidenced by the upregulation of several membrane receptors implicated in calcium-signaling cascades and intracellular calcium-mobilization and by the overrepresentation of NFAT1/Elk-1-binding sites in the promoters of the genes differentially expressed in these individuals. A coordinated upregulation of host genes associated with HIV-1 reverse transcription and viral transcription was also observed in EC-LTNPs –i.e. p21/CDKN1A, TNF, IER3 and GADD45B. We also found an upregulation of ANKRD54 in EC-LTNPs and viremic LTNPs in comparison with typical progressors and a clear alteration of type-I interferon signaling as a consequence of viremia in typical progressors before and after receiving antiretroviral therapy.

## Introduction

The chronic asymptomatic phase in HIV-1 pathogenesis is extremely variable, spanning from 2 to 25 years depending on the individual rate of disease progression defined by the interaction of host and viral factors^1,2^. However, a median time to AIDS since seroconversion between 8 and 11 years is generally accepted^3^. In order to categorize this variability, HIV-specialists have created a classification of HIV-1 infected individuals according to the disease progression, mainly measured by the loss of CD4^+^ T cells. In this sense, an extreme phenotype observed in long-term non-progressors (LTNPs) represents about 2% of all HIV-1 infected individuals and is characterized by the preservation of CD4^+^ T cell levels above 500 cells per µl of blood and relative low levels of viremia for at least ten years in the absence of ART^2^. Although some studies employ shorter periods of time to define LTNP condition, the use of 10 years of non-progression better differentiates between “true” LTNPs from those with delayed progression^4^. In parallel, some individuals called elite controllers (ECs) have the capacity to maintain undetectable levels of viral RNA without therapy for at least two years^5^. The prevalence of LTNPs and ECs have been determined in a huge military cohort of 4.586 naive HIV-1 infected individuals, representing the 2.04% and 0.55%, respectively^4^. The coexistence of EC and LTNP conditions observed in EC-LTNPs would represent the most beneficial host phenotype against HIV-1 infection, because of the capacity of these individuals to maintain elevated levels of CD4^+^ T cells and undetectable VLs over time^6^. This fact turns EC-LTNP phenotype into an interesting but infrequent group of study^7,8^.

EC-LTNP phenotype is considered a multifactorial phenomenon governed by viral fitness^9,10^ and host immuno-genetic mechanisms, such as CCR5Δ32 heterozygosity and the presence of HLA-B57/B27 and CCR2-V64I alleles^11–13^. Pereyra *et al*. have described that all the SNPs in the MHC associated with EC along with the genetic variants in CCR5 and CCR2 only explain 23% of the observed variance of durable host control, evidencing that these mechanisms are far away to fully explain the EC phenotype^14^. The existence of additional mechanisms has been studied even at transcriptome level employing microarray technologies^15,16^. However, hybridization-based methods have several limitations, such as hybridization specificity, background noise, hybridization to more than one gene product and a limited quantification range owing to signal saturation^17^. In contrast, RNA-Seq is a relatively recent application of high throughput sequencing technologies to transcriptome profiling^18^. Using this technology, the transcriptome of peripheral blood mononuclear cells (PBMCs) collected from EC-LTNPs has been characterized through the comparison with viremic LTNPs (vLTNPs) and HIV-positive individuals with a typical pattern of disease progression before (TP) and after receiving ART (TP-ART).

A global analysis of these transcriptomes was carried out in order to detect key transcripts to enable the classification of HIV-infected patients according to their phenotype and providing clues about the molecular mechanisms specifically associated with EC-LTNP phenotype through its comparison with vLTNP, TP and TP-ART. The understanding of viremia implications in remodeling the transcriptome machinery in patients on treatment was also investigated by the comparison of TP with TP-ART. The study of the genetic fingerprint exclusively found in EC-LTNPs would allow the molecular characterization of the most optimal immune activation against HIV-1 infection observed in nature and provides clues for the study of candidate markers for immunomodulatory drugs aiming at a functional HIV cure.

## Results

### Patients characteristics

A total of 23 patients were included in the study, seven patients with a typical pattern of disease progression and 16 patients with a LTNP phenotype as defined in materials and methods section. Typical progressors provided two different samples for the analysis, before ART (TP) in which a CD4^+^ T cell count depletion of 50–100 cells/mm^3^ per year along with a detectable viral load (VL > 5,000 copies/ml) were observed; and two years after ART treatment in which VL was under the level of detection (<20 RNA copies/ml; TP-ART). LTNP were classified as EC because of their undetectable viremia or detectable viremia with VL < 2,000 copies/ml in less than 25% of all determinations during the follow-up (EC-LTNPs). All EC-LTNPs (n = 8) showed a VL < 2,000 copies/ml at sampling time and three of them had undetectable viremia. The other 8 LTNPs showed detectable VLs < 10,000 copies/ml in more than 25% of all determinations during the follow-up and were considered as viremic LTNPs (vLTNPs). Patients’ characteristics are summarized in Table 1. No differences in gender, age or origin were found between groups of individuals. All LTNPs were followed for care for more than 10 years and all the individuals included in the study have a European ancestry and were diagnosed between 1988 and 1999.

**Table 1:** Main clinical characteristics of the patient groups included in the analysis.

Patient group	Gender		Population	Viral load in sample*	CD4 T cell count*
	Male	Female			
EC-LTNP	5 (62.5%)	3 (37.5%)	European	393 (Und.** − 1137)	667 (514–1081)
vLTNP	7 (87.5%)	1 (12.5%)	European	6021 (243–18900)	738 (492–1049)
TP	6 (85.7%)	1 (14.3%)	European	164900 (7620–585000)	302 (47–624)
TP-ART	6 (85.7%)	1 (14.3%)	European	Und.	554 (415–720)

*Mean (interval) **Und. = undetectable

### RNA-Seq quality control

A total of 30 cDNA libraries coming from EC-LTNP, vLTNP, TP and TP-ART were analyzed. There were on average 33,689,139 single-end reads per library and a mean of 30,854,460 reads per library were aligned to the human genome (91.6%) (Supplementary Fig. S1). Approximately, half of mapped reads aligned to each strand of the genome. A median above 32 of the Phred quality score was observed across all bases at each position of the 100 bp reads (Supplementary Fig. S1). A quality score above 32 indicates that the base-calling error probability was lower than 5.01 × 10^−4^. No statistically significant differences were identified between groups of HIV-positive individuals comparing the number of total and mapped reads.

### PBMC transcriptome profiling: global comparisons

**Figure 1:**
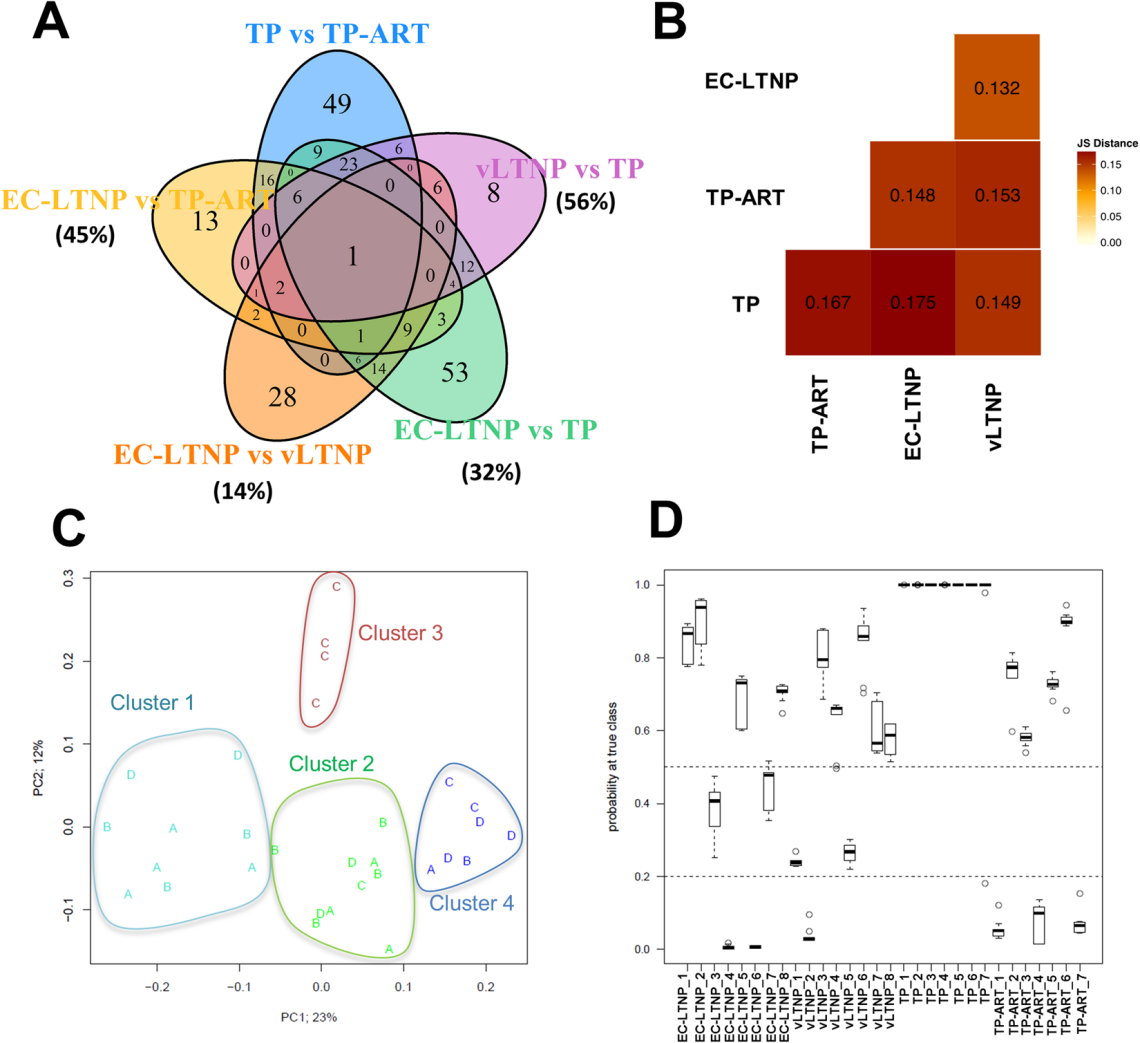
Comparison between phenotypes. Venn diagram showing overlapped DEGs found in several comparisons analyzed in the study (TP vs TP-ART, EC-LTNP vs TP-ART, EC-LTNP vs vLTNP, EC-LTNP vs TP and vLTNP vs TP). Only *SLC37A3* were found in all the comparisons analyzed (**A**). Distance matrix showing similarities between phenotypes, calculated by the Jensen-Shannon divergence as implemented in the Bioconductor’s package cummerbund (**B**). Multidimensional scaling (MDS) plot of the 30 samples based on the first two principal coordinates (PC, x and y axes). Labels A, B, C and D correspond to EC-LTNP, vLTNP, TP and TP-ART phenotypes, respectively. Color code is based on k-means clustering results with N = 4. The percentage of variability explained by each PC is indicated (**C**). Probabilities to be correctly classified for each individual employing the 20 best predictive genes. A total of ten independent predictions were carried out with LOOCV and the distribution of these probabilities are showed. The majority of the individuals (n = 22, 73.3%) were correctly classified and 20 of them obtained p-values > 0.5 at true class after 10 repetitions (and therefore p-values < 0.5 for the sum of the probabilities to be classified as any of the 3 other false classes). At the other extreme, some other individuals were repeatedly incorrectly classified with all the p-values < 0.2 for the 10 models. This is the case for EC-LTNPs 4 and 6, vLTNP 2 and TP-ARTs 1, 4 and 7. EC-LTNP 4 and 6 were classified as vLTNPs for all the repetitions whereas the vLTNP 2 was classified as EC-LTNP also in all the iterations. In the case of the three TP-ARTs erroneously classified (1, 4 and 7), two of them were classified as EC-LTNPs and the other one as vLTNP. Between these two situations, 2 individuals (vLTNPs 1 and 5) were ambiguously classified with p-values at true class below 0.3 and with similar p-values to be classified as EC-LTNPs. The 10 models were able to classify correctly all the TPs with p-values close to 1 in all cases (except 1 out of the 10 models for TP 7 which was classified as vLTNP) (**D**).

Different two by two comparisons were made between groups of patients. The number of differentially expressed genes (DEGs) were particularly high comparing EC-LTNPs with TP (n = 142) and TP with TP-ART (n = 119), suggesting the importance of an active viral replication in the modification of the transcriptome (Fig. 1A). According to the Jensen-Shannon distance based on the expression of these genes, the distance between EC-LTNPs and vLTNPs was lower than any other comparison (Fig. 1B). TP-ART are closer to EC-LTNPs than to themselves before ART (TP), supporting the hypotheses about the significance of the viremia in altering the expression of several genes in HIV-positive individuals. The effect of the viremia was measured in all the comparisons between groups of patients as the percent of DEGs observed in each condition which are coincident with the genes observed in TP versus TP-ART comparison. Thus, a 56%, 45% and 32% of DEGs found in vLTNP vs TP, EC-LTNP vs TP-ART and EC-LTNP vs TP comparisons, respectively, were also found in the TP versus TP-ART. However, only the 14% of DEGs found in EC-LTNP vs vLTNP were coincident with the genes found comparing TP and TP-ART (Fig. 1A).

### Selection of the most predictive genes of phenotypes

Gene expression variability was analyzed across the 30 samples through multi-dimensional scaling (MDS) and subsequent unbiased K-means clustering into four groups. Although the clustering of groups of patients was not achieved, each phenotype was preferentially represented in one of the clusters. We found EC-LTNPs preferentially in cluster 1, vLTNPs in cluster 2, TP in cluster 3 and TP-ART in cluster 4 (Fig. 1C). In total, 15 out of the 30 transcriptomes analyzed were correctly classified according to their corresponding phenotypes (50%). These results suggest a high level of heterogeneity within and between groups, complicating the finding of a common pattern of biomarkers associated to each phenotype.

**Figure 2:**
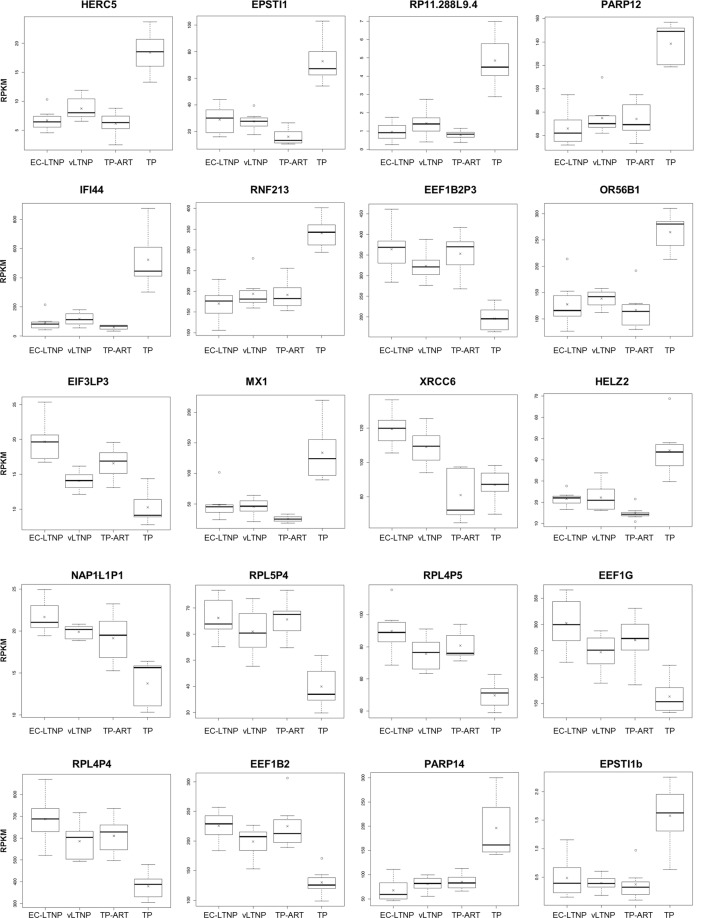
Best predictor genes of disease progression according to the hierarchical Bayesian classification model. The boxplots were generated in R and show the first and third quartile values for the RPKM distribution (upper and lower limits of the box), the median (the line splitting the box into two parts), the highest and lowest values (lines connected to the box through dashed lines), outlier values (open circles) and the mean value (crosses) for each phenotype.

New approximations such as the use of supervised classification techniques are necessary to describe new markers and mechanisms behind these phenotypes. Gene expression values from all the HIV-positive individuals included in the study were exposed to a gene selection process using a bias-corrected hierarchical Bayesian classification method. The expression values of a single panel of 20 genes were selected as the most accurate combination of genes to describe the phenotype variability found in the present study (Supplementary Fig. S2). The expression of most of these 20 genes is evidently different in TP compared with the other three phenotypes. However the expression of other genes such as *EIF3LP3* clearly distinguish phenotypes characterized by the control of viral replication (EC-LTNP + TP-ART) from phenotypes characterized by an active viral replication (vLTNP + TP) (Fig. 2). On his part, the expression of *XRCC6* clearly differentiates between phenotypes characterized by non-progression (EC-LTNP and vLTNP) and those characterized by a typical progression (TP and TP-ART) (Fig. 2).

Among the selected markers we found 13 genes, 6 pseudogenes with unknown function and a long intergenic non coding RNA (lincRNA) named RP11.288L9 as a negative regulator of IFI6. The functional annotation of these genes selected among the whole transcriptome identified several interferon-regulated genes (IRGs), including *HERC5*, *PARP12*, *IFI44*, *RNF213*, *MX1*, *HELZ2*, *EEF1G*, *EEF1B2*, *PARP14*, and *IFI6*. Several genes are related with RNA binding (*HERC5*, *EEF1G*, *EEF1B2*, *HELZ2*, *XRCC6*, and *PARP12*), response to virus (*IFI44*, *HERC5*, *EEF1G* and *MX1*), hydrolase activity (*HELZ2*, *XRCC6*, *RNF213* and *MX1*), eukaryotic translation elongation (*EEF1G*, *EEF1B2*, *and EEF1B2P3* as components of the eEF1 complex), NAD+ ADP-ribosyltransferase activity (*PARP12* and *PARP14*), and ribosomal activity (including the ribosomal protein pseudogenes *RPL5P4*, *RPL4P5* and *RPL4P4*).

### Phenotype prediction

A classification algorithm was created in order to evaluate the capacity of this panel of 20 genes to distinguish each phenotype. The distribution of probabilities to be correctly classified obtained in this algorithm for each individual was showed in Fig. 1D. The majority of the individuals (n = 22, 73.3%) were correctly classified (in contrast with the 50% obtained with the unbiased K-means clustering above described). Simplifying the model to only two phenotypes, LTNPs (regardless of their HIV-control capacity) and typical progressors (without considering if they are on ART or not), an accuracy of 90% (n = 27) was achieved (compared to the 76% obtained with the clustering). In this model, only three TP-ART individuals (TP-ART-1, TP-ART-4 and TP-ART-7) were incorrectly classified as LTNPs (Fig. 1D), suggesting that some of these mechanisms associated with virus control are common between LTNPs and individuals on therapy. Interestingly, these three samples erroneously classified as LTNP (TP-ART-1, TP-ART-4 and TP-ART7) were the TP-ART samples with higher CD4+ T cell counts (720, 650 and 578 cells per mm^3^, respectively compared with 525, 550, 439 and 415 cells per mm^3^ found in the rest TP-ART samples). The Matthews correlation coefficient (MCC) is used in machine learning as a measure of the quality of binary classifications. MCC ranges from −1 (total disagreement between prediction and observation) and +1 (perfect prediction), including 0 (no better than random prediction). The MCC obtained for the classification algorithm developed in the present study was 0.81, compared with the 0.53 obtained for the K-means clustering. These results support the use of supervised data mining classification methods combined with transcript abundance estimation as a promising approximation to characterize the transcriptome profile of a heterogeneous phenotype.

### Deregulation of type I interferon signaling as a consequence of viremia

**Figure 3:**
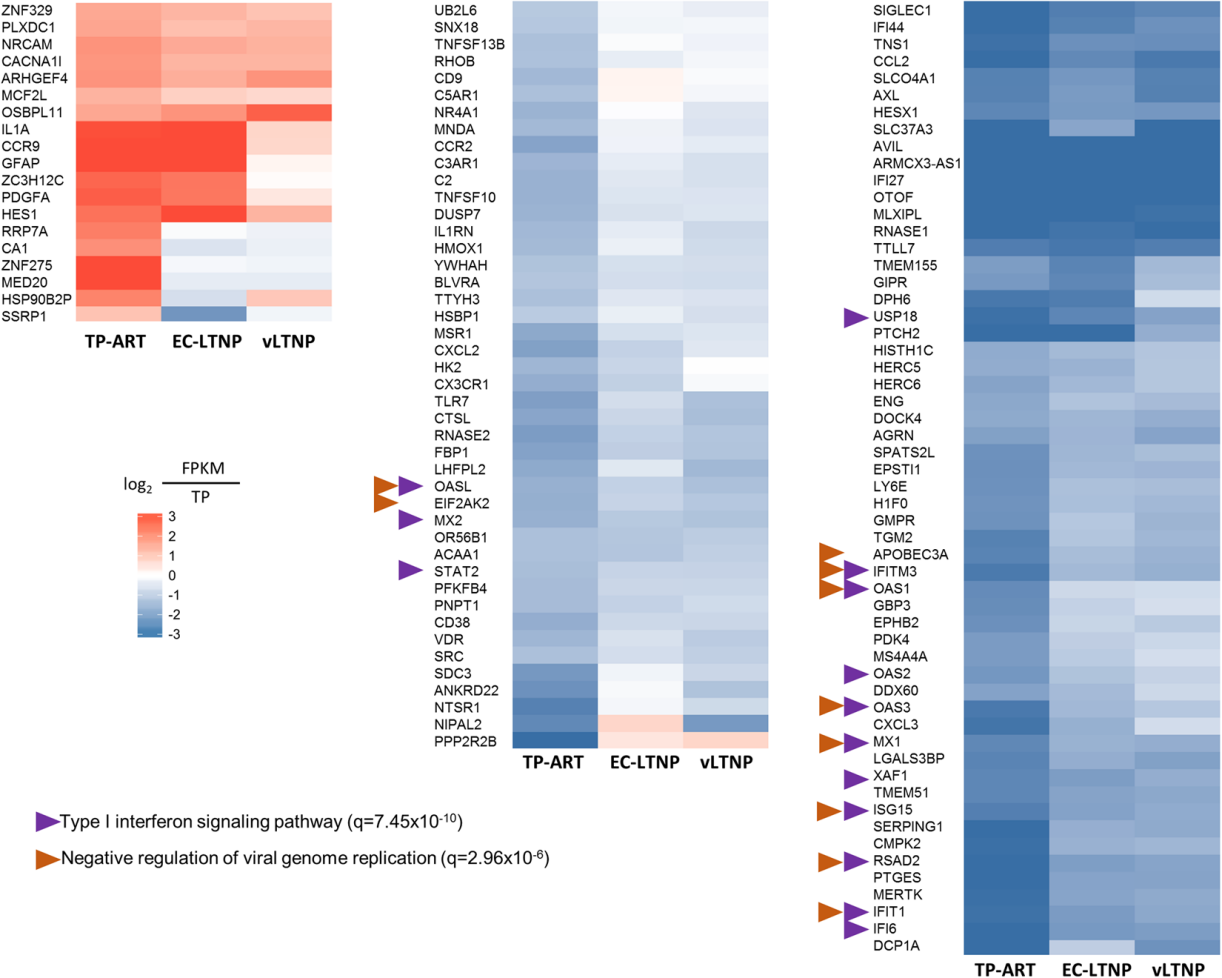
Deregulated genes in the TP/TP-ART comparison. Heatmap showing the comparison of the mean RPKM expression values for genes differentially expressed between TPs before and after receiving ART (the values for EC-LTNP and vLTNP were also showed just for the information). The RPKM expression values obtained for TPs, EC-LTNPs and vLTNPs are represented as a comparison with the values obtained for TPs.

The genes differentially expressed between any pair of phenotypes were identified using the negative binomial distribution. The expression of 119 genes was altered as a consequence of ART in TP individuals (Fig. 3). According to interferome there are evidences about the regulation of type I IFN in 92 out of these 119 genes (77.3%). The functional annotation of these 119 genes showed an enrichment of several molecular pathways related with interferon signaling and a defense response to virus (Supplementary Table S1).

Fourteen genes were included in Reactome’s type I interferon signaling pathway (q = 7.45 × 10^−10^). The expression of all these genes was downregulated after ART (Supplementary Fig. S3). The genes with higher differences between TP and TP-ART were the IRG *IFI27*, the antisense RNA *ARMCX3-AS1* and *ZNF275*. Five known anti-HIV IRGs were overexpressed in patients before ART including *EIF2AK2*, *ISG15*, *APOBEC3A*, *MX2* and *OAS1*, as well as other members of the OAS family, such as *OAS2*, *OAS3* and *OASL*. Five genes annotated as genes related to HIV-1 infection or resistance to AIDS were identified, including *CCL2*, *CX3CR1*, *TRIM22*, *SIGLEC1* and *TLR7*.

### EC-LTNP phenotype: activation of pathways leading to calcium release into the cytosol

**Figure 4:**
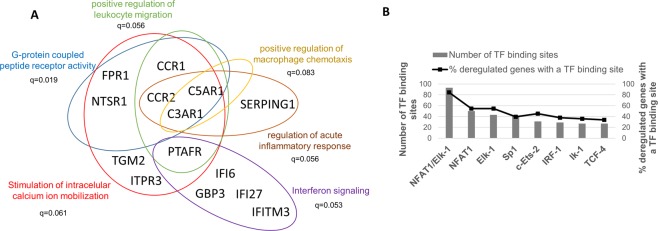
EC-LTNP versus TP-ART comparison. Gene Ontology terms statistically significant in the comparison EC-LTNP versus TP-ART (FDR corrected p-values < 0.1) (**A**). The promoter sequences of the genes differentially expressed between EC-LTNP and TP-ART were inspected to identify putative transcription factor binding sites using PROMO algorithm in ALGGEN server. The total number of transcriptional factor binding sites found and the percentage of these genes with a concrete binding site are showed (**B**).

The expression of 58 genes was deregulated in EC-LTNP compared with TP-ART (Supplementary Table S2). This comparison was selected in order to avoid the viremia as a confounding factor, since in both phenotypes control of HIV-1 replication is achieved either by treatment or spontaneously. Gene Ontology analysis revealed an enrichment of several genes implicated in G-protein coupled peptide receptor activity and the positive regulation of leukocyte migration. All these receptors are overexpressed in EC-LTNP and are related with the stimulation of intracellular calcium ion mobilization (Fig. 4A).

The promotor sequence of the 58 genes deregulated in EC-LTNP compared with TP-ART were analyzed to identify transcription factor binding sites (TFBS). The analysis of the TFBS showed that NFAT1 binding site was the most frequent within the promotor of these genes (50 NFAT1-binding sites) and was predicted within the promoter of 29 out of the 58 (50%) differentially expressed genes (Fig. 4B and Supplementary Table S3). Forty-three Elk-1 TFBS were also found in 29 out of the 58 genes (50%) (Fig. 4B). The overall genes with an NFAT1 or Elk-1 binding site in their promoter regions are 49 (84.5%). All these results suggest that a different regulation of the intracellular calcium signaling is observed in EC-LTNPs compared with TP-ART.

### Molecular mechanisms involved in the control of HIV-1 replication in LTNP

**Figure 5:**
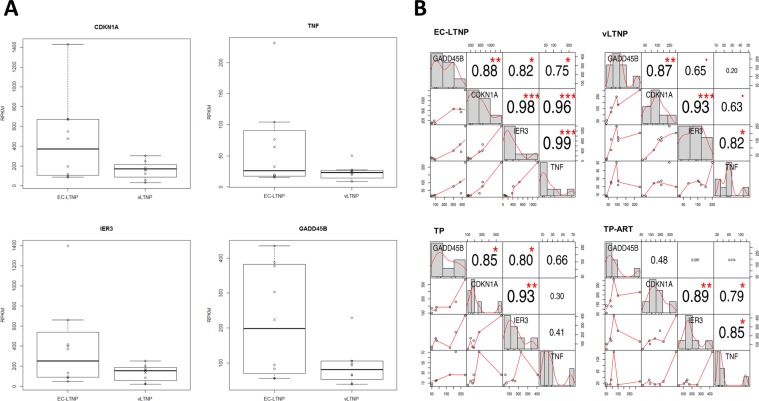
Expression of *GADD45B*, *CDKN1A*, *IER3* and *TNF* genes. RPKMs obtained for each gene in EC-LTNPs and vLTNPS (**A**). The correlation of these expression values between genes is shown for each group of patients. The distribution of each variable is shown on the diagonal, the bivariate scatter plots of RPKMs with a fitted line are displayed on the bottom of the diagonal and the value of the correlation plus the significance level as stars on the top of the diagonal according to Pearson parametric correlation test. This plot was generated using “PerformanceAnalytics” R package. Statistically significant p- values indicate a significant linear relationship between the expression values of two genes and are displayed as follows: ***p < 0.001; **p < 0.01 and *p < 0.05 (**B**).

The expression of 70 genes was altered in EC-LTNPs in comparison with vLTNPs (Supplementary Table S4). The functional annotation of these genes showed no enrichment of any specific pathway or GO term. Some of these genes were key genes related to host mechanisms leading to modify HIV-1 replication and transcription, including the upregulation of *CDKN1A* (encoding the cyclin dependent kinase p21), TNF and the TNF-network associated gene *IER3*, and *GADD45B* (Fig. 5A). The expression of these four genes was highly correlated in EC-LTNP (statistically significant correlations), but was not in the other phenotypes (Fig. 5B).

**Figure 6:**
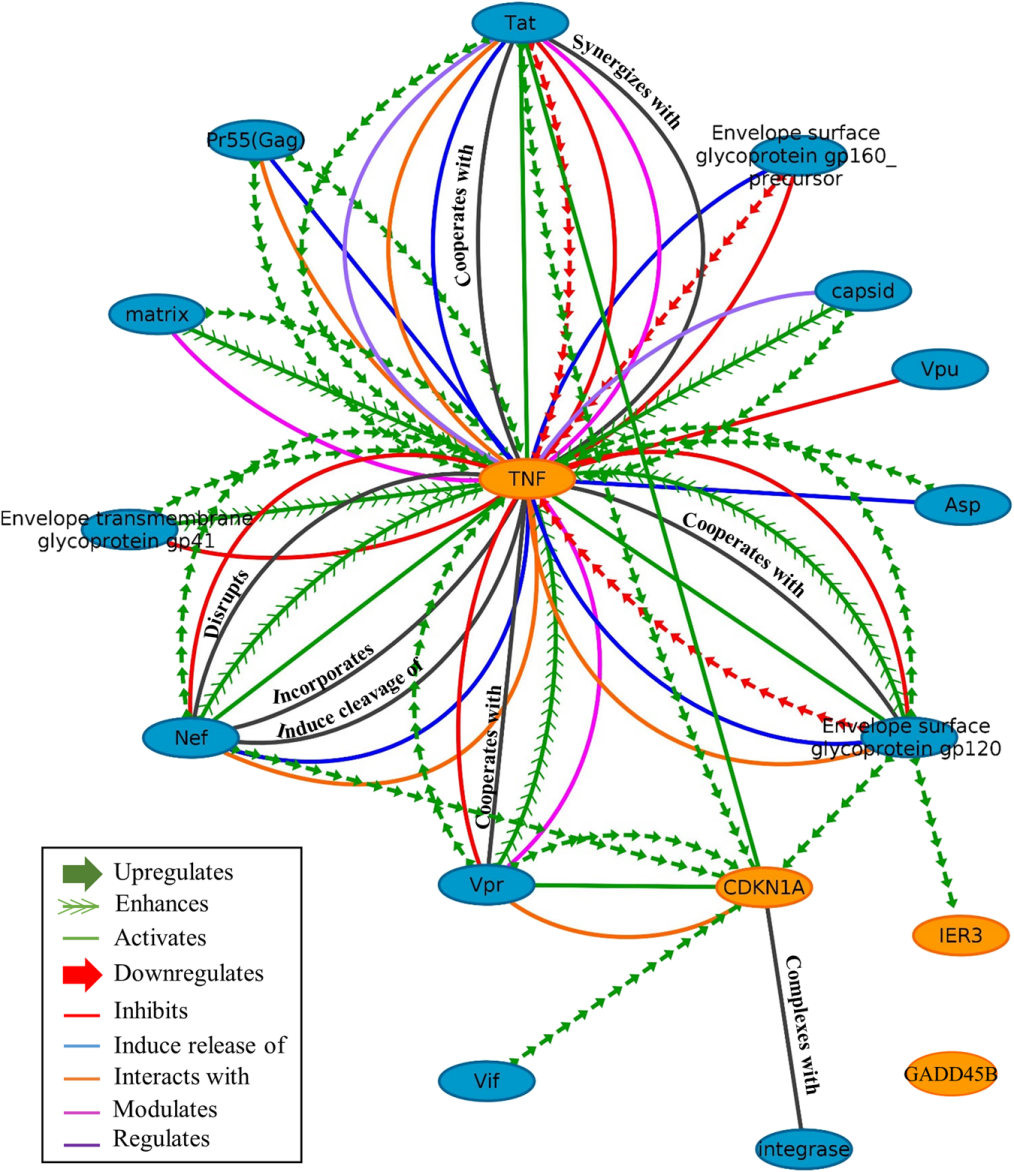
Protein-protein interaction network of CDKN1A, TNF, IER3 and GADD45B with viral proteins. The whole HIV-1 human interaction database was downloaded and all the interactions of CDKN1A, TNF, IER3 and GADD45B with viral proteins were mapped.

According to the HIV-1 Human Interaction Database, there are evidences of 64 non-redundant interactions between these 4 proteins (CDKN1A, TNF, IER3 and GADD45B) and 13 HIV-1 proteins (Fig. 6). These results suggest that the expression of CDKN1A, TNF, IER3 and GADD45B are optimally coordinated in EC-LTNPs to regulate the expression of viral proteins.

### Genes associated with LTNP phenotype

**Figure 7:**
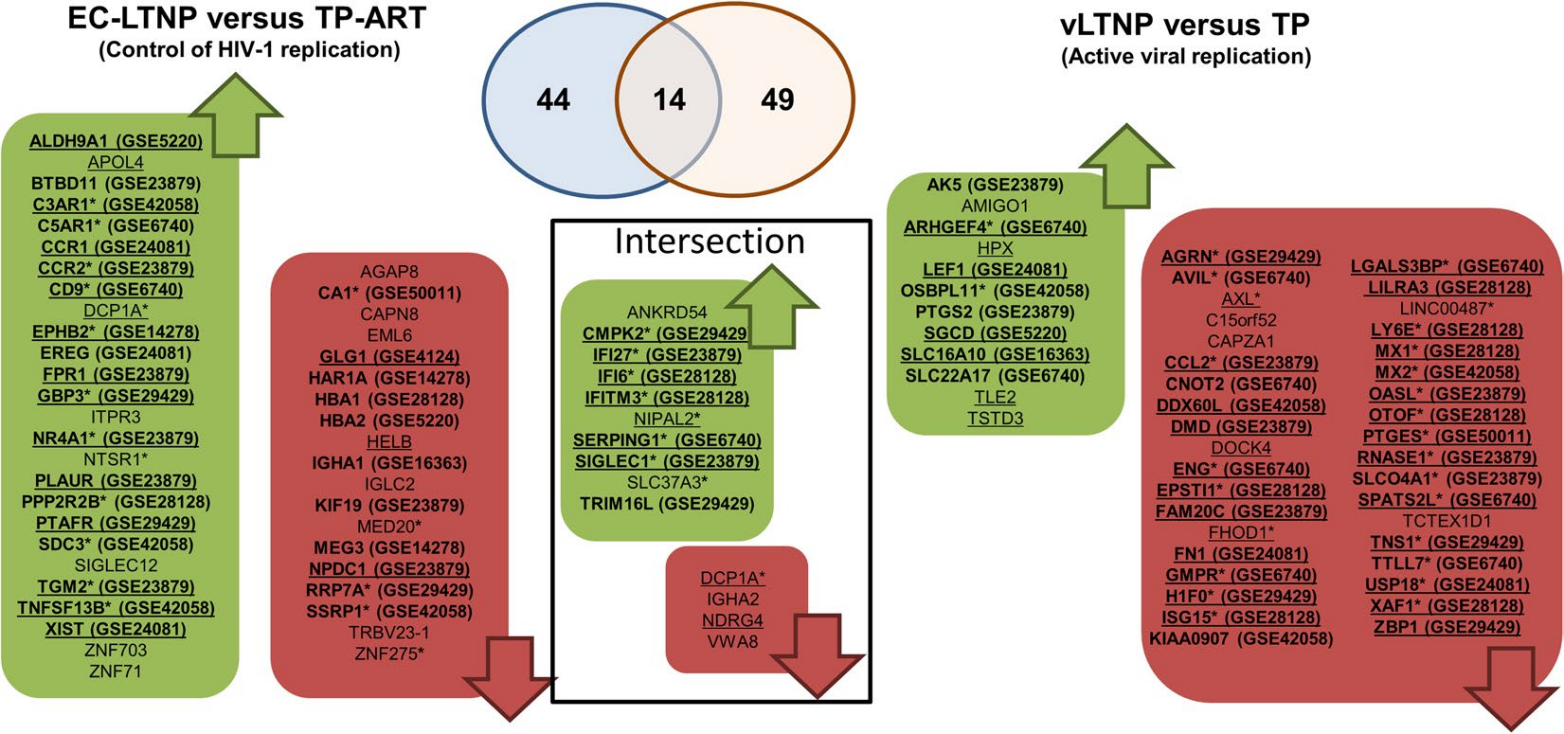
Genes associated with LTNP condition. The figure shows DEGs in the EC-LTNP versus TP-ART and vLTNP versus TP comparisons. Framed genes represent the intersection between both comparisons. Green and red boxes show upregulated and downregulated DEGs, respectively. Genes regulated by interferon are underlined whereas genes associated with an active viral replication (identified by the comparison of TP with TP-ART) are designated by an asterisk. DEGs found in other transcriptomic profiling studies of HIV-positive individuals with different degrees of disease progression were searched for a curated dataset collection^54^. The DEGs found in other datasets are in bold, indicating their Gene Expression Omnibus (GEO) accession numbers in parenthesis. Only datasets generated from blood cells and with a fold change of at least 2.0 were included in this figure. The included datasets and the phenotypes compared were: GSE14278 (HIV resistent vs HIV high-risk negative), GSE16363 (aviremic vs viremic), GSE23879 (elite controller vs HIV-negative), GSE24081 (controller vs progressor), GSE28128 (CD4 rapid progressors vs CD8 rapid progressors), GSE29429 (healthy vs HIV-positive), GSE4124 (HIV− vs HIV+ transmitter), GSE42058 (uninfected vs HIV infected), GSE50011 (CD4 count >500 vs CD4 count <500), GSE5220 (aviremic vs viremic), GSE6740 (CD4 uninfected vs CD4 non-progressor) and GSE6740 (CD8 non-progressor vs CD8 acute).

Common markers to all the LTNPs (EC-LTNP and vLTNP) were investigated. In order to minimize the effect of an active viral replication on the transcriptome profiles, two independent comparisons were simultaneously carried out, EC-LTNP versus TP-ART (as previously mentioned) and vLTNP versus TP, comparing two phenotypes with an active viral replication. The expression of 58 genes was dysregulated in EC-LTNPs compared with TP-ART, whereas 63 were identified in the vLTNP versus TP analysis (Fig. 7). A total of 14 genes were identified in both comparisons (EC-LTNP/TP-ART and vLTNP/TP; Fig. 7). Among these 14 genes, 9 were identified as IRGs and only *ANKRD54*, *IGHA2* and *VWA8* were not associated with the viremia in the comparison of TP versus TP-ART. A downregulation of the Von Willebrand Factor A Domain Containing 8 (VWA8) and the constant region of the heavy chain of IgA2 (IGHA2) was observed in LTNPs. Of note, only ANKRD54 were found strongly upregulated in both EC-LTNP and vLTNP with respect typical progressors with fold changes over 25.

## Discussion

As expected from the multifactorial nature of HIV disease progression, the translation of the phenotype differences observed in EC-LTNP, vLTNP, TP and TP-ART to transcriptome differences does not seem to be obvious. A clear clustering of the individuals with different patterns of disease progression was not observed, evidencing the necessity to apply different approximations to define a group of biomarkers to better differentiate between groups of individuals. Machine learning techniques such as supervised classification are designed to analyze large amounts of data and infer a function from a training data set with several predictive variables (mRNA expression values) associated to a known output (phenotype). A mathematical model able to distinguish between LTNPs (regardless of their HIV-control capacity) and TPs (without considering if they are on ART or not) with an accuracy of 90% was obtained using hierarchical Bayesian classification algorithms combined with the selection of 20 genes as the best predictors of HIV disease progression. Several of these 20 genes were IRGs, pointing to the importance of the interferon regulation in HIV disease progression. As expected, the majority of these IRGs were downregulated in patients with low/undetectable levels of viremia. On the contrary, a particular upregulation of other IRGs implicated in reverse transcription and transcription of viral genes were especially observed in EC-LTNPs. First, an upregulation of the eukaryotic translation elongation factors of the eEF1 complex (EEF1G, EEF1B2 and EEF1B2P3), considered critical HIV-1 reverse transcription cofactors^19^. Second, an upregulation of XRCC6 which associates with Tat and TAR and repress the transcription of viral mRNAs^20^. Third, the downregulation of a lincRNA located within the promotor of IFI6 gene and implicated in the negative regulation of IFI6^21^. This methodology represents an alternative way to associate differences in gene expression with a concrete phenotype, combining a solid transcript abundance estimation procedure (Tophat/Cufflinks) with machine learning approaches.

Deciphering the molecular mechanisms responsible for the control of HIV-1 replication for long periods of time observed in EC-LTNPs is absolutely crucial to mimic this spontaneous defense against the virus in HIV vaccinology and functional cure strategies. Different mechanisms related with reverse transcription and viral transcription have been specifically detected in EC-LTNP, which is consistent with the functional annotation obtained for the genes selected as the best predictors of disease progression. HIV-1 reverse transcription depends on the phosphorylation of viral reverse transcriptase by a host kinase named CDK2. Viral reverse transcriptase phosphorylation at a conserved threonine by CDK2 increases its efficacy and stability and enhances its viral fitness^22^. This mechanism is regulated by the cyclin-dependent kinase inhibitor 1A (CDKN1A)-mediated inhibition of CDK2 and has been previously described in individuals with EC phenotype^22^. In this study an upregulation of *CDKN1A* in EC-LTNP individuals compared with vLTNP was observed, suggesting an inactivation of CDK2 activity, avoiding the phosphorylation of viral reverse transcriptase and diminishing its efficacy and stability^23^. Moreover, a second mechanism related with CDKN1A has been associated with EC phenotype, describing a partial resistance of CD4^+^ T cells from these individuals to HIV-1 infection mediated by a strong and selective upregulation of CDKN1A, also called p21^24^. This mechanism seems to regulate viral mRNA elongation by inactivating the enzymatic activity of CDK9, essential for the proper elongation of HIV-1 mRNA as a component of the P-TEFb (positive transcription elongation factor) complex^25^. This P-TEFb complex is formed by CDK9 and Cyclin T1 and is recruited after the activation of the viral LTR activity as a consequence of HIV-1 Tat protein binding to the trans-activation response (TAR) RNA structure. This mechanism is responsible for Tat-activated transcriptional elongation of viral transcripts^26^.

Aside from CDKN1A, other genes related with HIV-1 mRNA elongation were upregulated in EC-LTNP compared with vLTNP, including TNF, immediate early response 3 (IER3) and growth arrest and DNA damage 45 (GADD45B). GADD45B contribute to apoptosis and regulate HIV transcription^27^. GADD45B inhibits HIV-1 gene expression independent of CDKN1A, apparently without the need of the TAR, NF-κB, NRE and SP1 sites^27^. IER3 inhibits the most important family of Ser/Thr phosphatases, the protein phosphatase 2 A (PP2A), which in turn induces basal but not the Tat-activated HIV-1 transcription^28^. The functional implication of TNF in HIV-1 transcription is linked to its role in regulating IER3 gene expression^29^. One important finding in our work is the high correlation of the expression of CDKN1A, IER3, GADD45B and TNF found in EC-LTNPs compared with the other phenotypes. These results along with the analysis of the human protein interaction network with viral proteins suggest that these genes are coordinately and optimally regulated in these individuals to modulate basal and Tat-activated HIV-1 transcription.

We have also found evidences of an altered regulation of calcium-dependent signaling cascades in EC-LTNP compared with TP-ART, which is consistent with the capacity of these individuals to control viral transcription^30^. First, an enrichment in EC-LTNP of cell surface receptors involved in the stimulation of intracellular calcium mobilization directly (FPR1, CCR2) or by the activation of a phosphatidylinositol-calcium second messenger system (NTSR1). Second, a strong upregulation of inositol triphosphate (IP3) receptor isoform 3 (ITPR3) which mediates the mobilization of calcium ions into the cytosol in response to IP3. Third, an overrepresentation of the nuclear factor of activated T cells (NFAT1) and Elk-1 binding sites in the promotors of the genes differentially expressed. NFAT1 is present in the cytosol and are dephosphorylated by the Ca^2+^/calmodulin-dependent phosphatase calcineurin as a response to increased concentrations of intracellular Ca^2+^, causing a conformational change that results in its translocation from the cytoplasm to the nucleus and the activation of the transcription of NFAT1 target genes^31^. The stimulation of calcium-sensing receptors has also been associated with the activation of the transcription factor Elk-1 and the subsequent regulation of gene transcription^32^. Four, the increased expression in EC-LTNPs of several genes implicated in the positive regulation of the cytosolic calcium ion concentration including CXCL10, CCR9, ITPR3 and S1PR3, suggesting an additive effect on EC-LTNP over vLTNP.

Acute HIV-1 infection is characterized by a compartmentalized CD4^+^ T cell depletion and constant viral replication, counteracted by a broad antiviral effect of the innate immune response. Type I IFNs play a leading role in this process through the activation of hundreds of IRGs. Little is known about the transcriptome changes experienced by HIV-1 infected individuals before and after receiving ART. The experimental design of the present study has allowed the identification of deregulated genes as a consequence of viremia, detecting altered mRNA levels of 119 genes, mostly regulated by interferon. The upregulation of these IRGs in patients with detectable HIV-1 replication and a typical rate of disease progression demonstrates the tight relationship between the pathogenesis of HIV infection and the chronic IFN stimulation^33,34^. These genes are mainly involved in the general immune response against viral infections such as OAS and MX genes^35^. Of note, some of these genes have been directly related with HIV pathogenesis, including *IFI27*, *IFITM3* and *TRIM22*^36–38^. The higher difference found comparing HIV-infected individuals before and after the treatment was the mRNA levels of the interferon alpha-inducible protein IFI27.

Comparing EC-LTNP vs TP-ART and vLTNP vs TP, several genes were found in both comparisons associated to both LTNP phenotypes –i.e. EC-LTNPs and vLTNPs, but only three DEGs were not affected by the viremia according to the TP versus TP-ART comparison. Thus, similar levels of these three genes (*ANKRD54*, *IGHA2* and *VWA8*) were found in all LTNPs (EC-LTNPs and vLTNPs). Neither were regulated by interferon. *ANKRD54* encodes an ankyrin repeat containing protein upregulated in LTNPs. Some proteins containing this type of ankyrin repeats interact with HIV-1 proteins such as Vpr^39^. Artificial ankyrins have been designed targeting the capsid domain of the HIV-1 Gag polyprotein, showing an antiviral effect at post-integration steps and inhibiting the virus assembly and egress pathway^40^. The role of this potential molecular marker of disease progression naturally overexpressed in LTNPs should be further investigated.

One limitation of the current study is that transcriptome was determined in total PBMCs and was not in specific lymphoid subpopulations. Nevertheless, these samples reflect different immune environments, such as possible differences in the relative proportions of cell types implicated in the immunopathogenesis of HIV-1 infection, i.e. resting CD4^+^ T cells. This information is complementary and compatible with the study of specific subpopulations. However, the characterization of the transcriptome of a single cell can be achieved thanks to the advance in second and third generation sequencing technologies along with the relatively recent establishment of procedures to synthesize double-stranded cDNA from the extremely low quantities of mRNA present in a single cell, or even in a single nucleus^41^. Single cell RNA-Seq analyses will allow a deeper and definitive characterization of the immune response against HIV-1 infection observed in EC-LTNPs, identifying unequivocally which cell types are responsible for the expression of which host restriction factors. The present study exposes the changes in the transcriptome associated with different patterns of disease progression observed in HIV-positive individuals. Specifically, the analysis of EC-LTNPs as the most beneficial phenotype of immune activation against HIV-1 infection has allowed the identification of deregulated expression levels of several molecules in these patients. We propose that the coordinated upregulation of *CDKN1A*, *IER3*, *GADD45B* and *TNF* as well as the positive regulation of calcium-dependent signaling could be involved in the mechanisms leading to the slower progression to AIDS and HIV control concomitantly observed in EC-LTNPs.

## Material and Methods

### Study population

Samples from patients were kindly provided by the HIV BioBank integrated in the Spanish Research Network (RIS). Samples were processed following current procedures and frozen at −80 °C immediately after their reception. All patients participating in the study gave their informed consent and protocols were approved by Institutional Ethical Committees (Instituto de Salud Carlos III. CEI PI 10_2011v3). A total of 30 cDNA libraries were analyzed, including those coming from 16 HIV-positive individuals who have been classified as LTNPs within the Spanish LTNP-RIS cohort, 7 HIV-positive patients with a typical pattern of disease progression before ART (TPs) and the same 7 individuals after receiving ART (TPs-ART) from the Spanish CoRIS cohort. All the LTNP individuals maintain CD4^+^ T cell counts over 500 cells per mm^3^ and a VL under 10,000 copies per ml of blood in all the VL determinations during the first 10 years from infection/HIV^+^ diagnosis. All experiments were performed in accordance with relevant guidelines and regulations.

### RNA extraction, mRNA library preparation and sequencing

Total RNA from all the HIV-infected individuals was extracted from 10^7^ peripheral blood mononuclear cells (PBMCs) with mRNeasy Mini Kit (Qiagen) obtaining 5–20 µg of total RNA. The quality of the RNA was measured in a 2100 Bioanalyzer (Agilent Technologies), obtaining a mean RNA integrity number (RIN) value of 9.2, with RIN values greater than 8 for all samples. The cDNA libraries from total RNA samples were prepared by an Illumina TruSeq RNA sample prep kit (Illumina, San Diego, CA) and were clustered onto a TruSeq single-end flow cell using a TruSeq SR Cluster Kit v3-cBot-HS (Illumina, San Diego, CA), after quantification by PicoGreen dsDNA assay kit (Life Technologies) and pooling in equimolar mixtures. Finally, the DNA sequence of each cluster on flow cells was determined employing 100 cycles of Sequencing-By-Synthesis (SBS) technology (TruSeq SBS Kit v3-HS kit) on an Illumina’s HiSeq2000 Sequencing System.

### Analysis of sequencing data

A first quality assessment was performed with FastQC and reads were trimmed with the java tool Trimmomatic with default paramenters in order to remove sequences of primers and adapters employed during library preparation from the ends of sequences. The adapters and Illumina-specific primers from the reads were removed, allowing two mismatches, requiring a minimum of 30 matches in palindromic mode and a minimum of ten matches for nonpalindromic mode between the read sequence and the adapters/primers. A sliding window of 4 nucleotides were analyzed for each read and were trimmed once the average Phred quality falls below 30. The end bases below a quality score of 3 were cut. Sequences trimmed to shorter than 60 bases were removed.

Filtered reads were aligned to the human genome assembly GRCh37 using Bowtie2/Tophat2^42,43^ and the transcript assembly was reconstructed with Cufflinks^44,45^. Differential expression analysis was carried out with Cuffdiff package based on the negative binomial distribution. Cuffdiff transforms these gene counts to units of reads per kilobase of transcript length per million mapped reads (RPKMs) and infers probabilities before (p-values) and after FDR correction (q-values) to identify differentially expressed genes among groups of individuals (q-values < 0.05). The similarities between phenotypes were analyzed through the calculation of Jensen-Shannon distance with the Bioconductor package cummeRbund. The variability found between transcriptome profiles was explored in R software through K means clustering and dimensionality reduction by MDS based on the Pearson correlation using “stats” and “gplots” packages.

The genes differentially expressed between EC-LTNPs, vLTNPs, TPs and TP-ARTs were identified for each possible dual comparison between any pair of the above mentioned phenotypes (6 comparisons in total). This type of analysis identified genes differentially expressed in individuals exhibiting an active HIV-1 replication (vLTNPs and TPs) compared with individuals with a controlled viremia (EC-LTNPs and TP-ARTs). This approximation also analyze the distinguishing factors between EC-LTNPs and vLTNPs and the identification of the main alterations at the transcriptome level as a consequence of ART administration through the comparison of the same individuals before and after receiving anti-HIV therapy.

### Predictive genes of disease progression by data mining techniques

The predictive model of disease progression was created combining the transcript abundance estimation obtained from Cuffdiff with a bias-corrected feature selection procedure and a hierarchical Bayesian classification^46,47^. The RPKMs obtained for every single gene and for all the individuals were subjected to a wrapper feature selection process and a subsequent supervised classification by a hierarchical Bayesian classification. During this process, the more informative combination of transcripts to define the progression phenotype were selected. Genes with expression values with near zero variance were eliminated prior modeling with the default parameters of nearZeroVar function included in “caret” Bioconductor package. The feature selection process of the remaining genes included the same algorithm used for classification to evaluate the importance of each gene through 10 repetitions of leave one out cross-validation (LOOCV), setting up random seeds for each repetition^46^. Two criteria were employed to evaluate the optimal selection of predictive genes, including ER and AMLP (which evaluate the classification models more accurately by taking into account low predictive probabilities at the true class labels^47^). The smaller subset of genes reaching the lower ER and AMLP in this process was selected for the final classification model. The probability for each individual to be classified as EC-LTNP, vLTNP, TP or TP-ART were calculated by the classification model using the estimated expression values of selected transcipts. The RPKMs values obtained for each individual were obtained from the output of Cuffdiff tool (genes.read_group_tracking file) and the Bioconductor packages “caret” and “BCBCSF” were employed for pre-processing, feature selection and classification processes^47^.

### Functional annotation

The identification of enriched biological pathways, diseases or gene ontology terms associated with differentially expressed genes between groups of patients was carried out with KOBAS 2.0 tool^48^. This software integrates searches against the main biological databases, including Gene Ontology (GO), KEGG, PID, Reactome, PANTHER, GO, IMIM, FunDO, GAD and NHGRI GWAS Catalog. Interferome v2.01 was used to find evidences about interferon regulation of genes deregulated in any of the comparisons carried out in the present study^49^.

### Identification of putative transcription factor binding sites

A 1500 base pairs sequence upstream of the start codon of the genes was retrieved from GRCh38 using Ensembl^50^. These sequences were inspected to identify putative transcription factor binding sites included in TRANSFAC database using PROMO algorithm in ALGGEN server^51^. The reliability of this methodology was calculated through the expectation of finding each binding site in a random sequence of 1000 nucleotides, considering a model with exactly the same nucleotide frequency as the query sequence (E-value). A very conservative cutoff was used to predict the transcription factor binding sites (TFBS) and only TFBS with an E-value < 0.05 and with a similarity to the matrix >95% were considered as true binding sites.

### Interactions of human proteins with HIV-1 proteins

We downloaded the whole database of HIV-1 and human protein interactions^52^. The HIV-human protein interaction network was visualized in Cytoscape_v3.1.1^53^. We mapped all the interactions of CDKN1A, IER3, GADD45B and TNF with viral proteins, distinguishing the type of interaction (mainly upregulation, activation, enhancement, downregulation and inhibition).

## Supplementary information

Supplementary Figures

Supplementary Table S1

Supplementary Table S2

Supplementary Table S3

Supplementary Table S4

## Data Availability

The access to the raw reads for use by the scientific community can be done upon request to the authors and after approval of every single request by the Data Protection Officer of the Instituto de Salud Carlos III.
