# Supplementary material for: Transcriptome Sequencing of Peripheral Blood Mononuclear Cells from Elite Controller-Long Term Non Progressors
Source: Sci Rep. 2019 Oct 3;9:14265. doi: 10.1038/s41598-019-50642-x (PMC6776652; doi:10.1038/s41598-019-50642-x)
Supplement: Supplementary file 1 — Supplementary Figures [file 41598_2019_50642_MOESM1_ESM.pdf]

TRANSCRIPTOME SEQUENCING OF PERIPHERAL BLOOD MONONUCLEAR CELLS FROM ELITE CONTROLLER-LONG TERM NON PROGRESSORS

Francisco Díez-Fuertes, Humberto Erick De La Torre-Tarazona, Esther Calonge, Maria Pernas, María del Mar Alonso-Socas, Laura Capa, Javier García-Pérez, Anavaj Sakuntabhai, José Alcamí

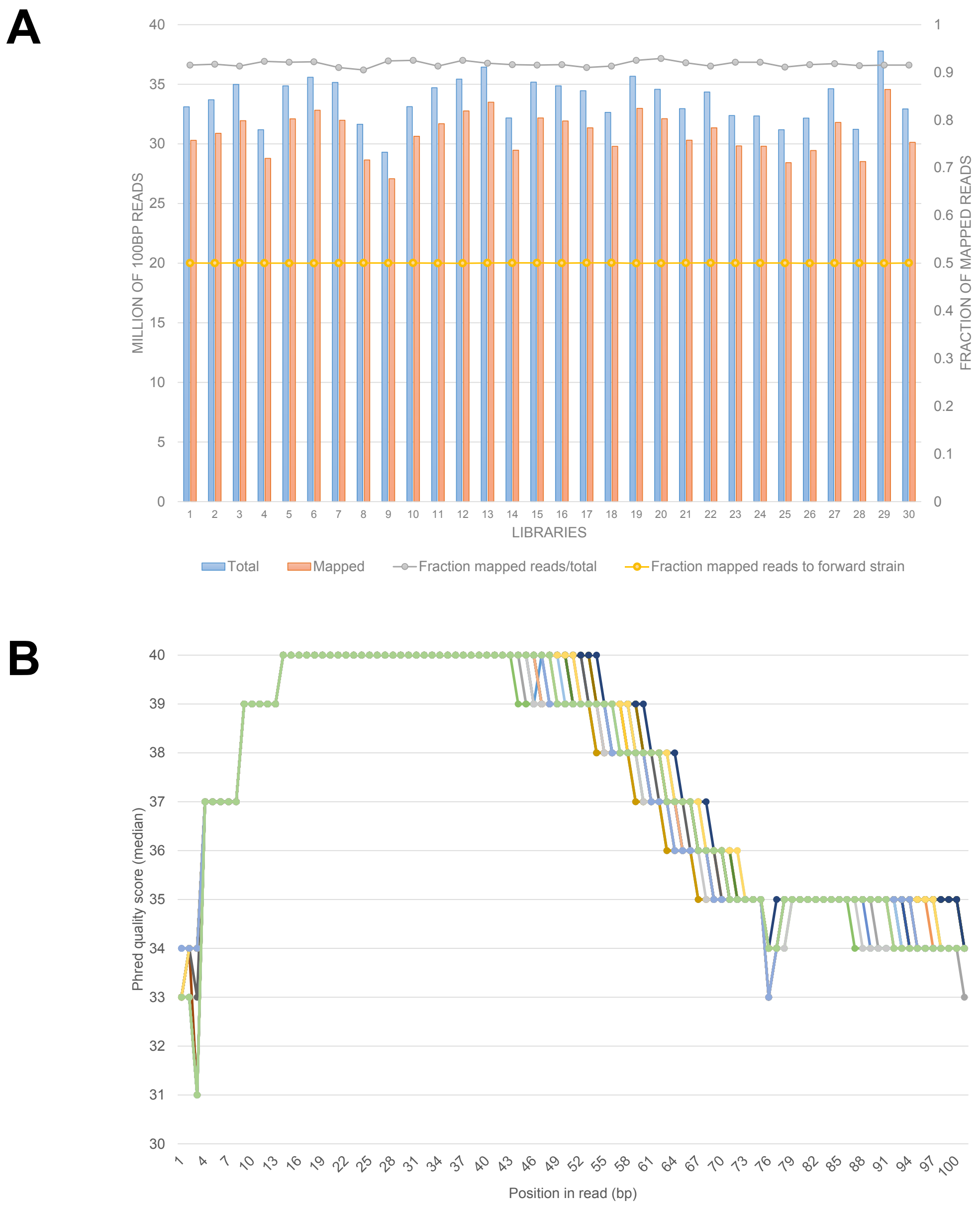

Supplementary Figure S1. Reads per library obtained by RNA-seq and quality control. Number of total reads obtained for each library and reads mapped to human genome, including the fraction of mapped/total reads and the fraction of mapped reads to forward strain (A). Median of the Phred quality score for the 30 libraries included in the study (B).

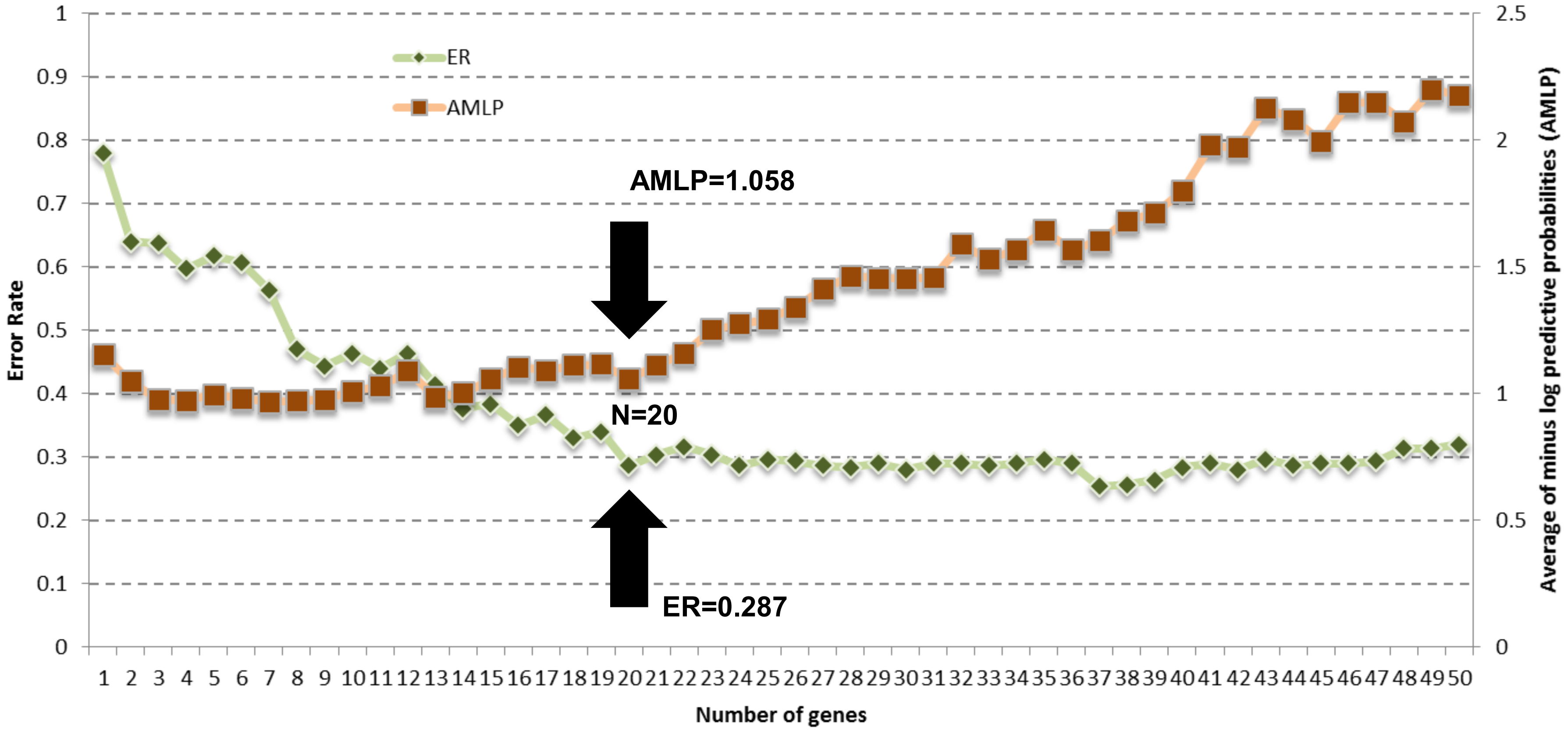

Supplementary Figure S2. Identification of the best predictor genes of patient phenotype. Error rate (ER) and average minus log predictive probabilities (AMLP) obtained in the feature selection process, evaluating the accuracy of 50 models employing the 1-50 best predictive genes. The model with 20 predictive genes was selected as the most accurate model.

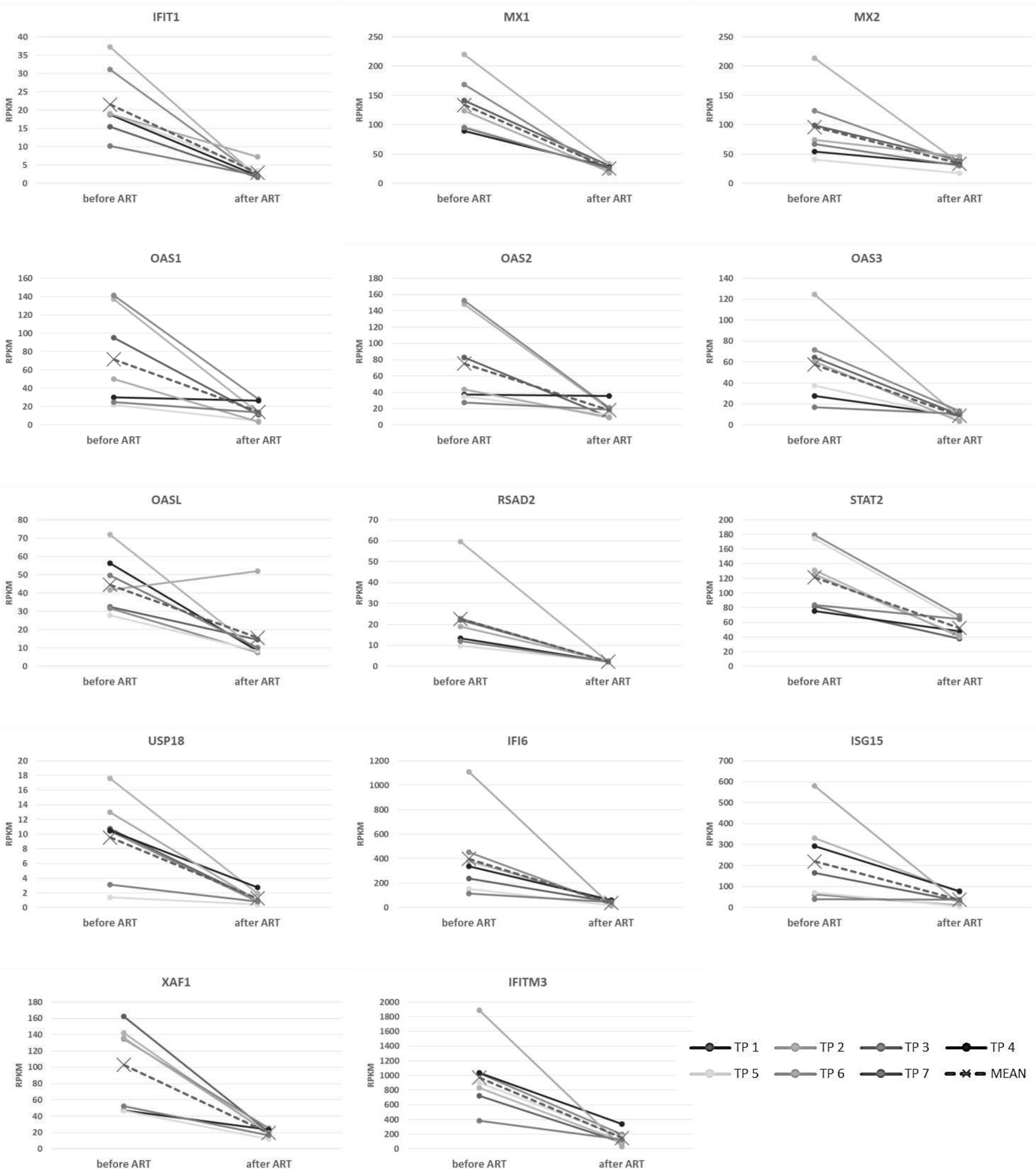

Supplementary Figure S3. **Restoration of IRGs expression as a consequence of ART in typical progressors**. RPKM values obtained for the 20 IRGs differentially expressed in TPs and TPs and for each patient before and after receiving ART showing evidences about type I IFN regulation in humans.
